# Potential adverse drug events and its predictors among hospitalized patients at medical center in Ethiopia: a prospective observational study

**DOI:** 10.1038/s41598-021-91281-5

**Published:** 2021-06-03

**Authors:** Tamiru Sahilu, Mestawet Getachew, Tsegaye Melaku, Tadesse Sheleme, Duresa Abu, Tesfu Zewdu

**Affiliations:** 1 Department of Pharmacy, College of Health Science, Assosa University, Assosa, Ethiopia; 2 Department of Clinical Pharmacy, School of Pharmacy, Institute of Health, Jimma University, Jimma, Ethiopia; 3 Department of Pharmacy, College of Public Health and Medical Science, Mettu University, Metu Zuria, Ethiopia; 4 Department of Nursing, College of Health Science, Assosa University, Assosa, Ethiopia

**Keywords:** Diseases, Health care, Medical research, Risk factors

## Abstract

Potential adverse drug event (PADE) is a medication error with the potential to cause associate degree injury however that does not cause any injury, either due to specific circumstances, chance, or as a result of the error being intercepted and corrected. This study aimed to assess the incidence, contributing factors, predictors, severity, and preventability of PADEs among hospitalized adult patients at Jimma Medical Center. A prospective observational study was conducted among hospitalized adult patients at a tertiary hospital in Ethiopia. Logistic regression was performed to identify factors predicting PADE occurrence. P-value < 0.05 was considered for statistical significance. A total of 319 patients were included. About 50.5% of them were females. The mean ± SD age of the participants was 43 ± 17.6 years. Ninety-four PADEs were identified. Number of medications (adjusted OR = 5.12; 95% CI: 2.01–13.05; p = 0.001), anticoagulants (adjusted OR = 2.51; 95% CI: 1.22–5.19; p = 0.013), anti-seizures (adjusted OR = 21.96; 95% CI: 6.57–73.39; p < 0.0001), anti-tuberculosis (adjusted OR = 2.2; 95% CI: 1.002–4.59, p = 0.049), and Elixhauser comorbidity Index ≤ 15 (adjusted OR = 6.24; 95% CI: 1.48–26.25, p = 0.013) were independent predictors of PADEs occurrence. About one-third of patients admitted to the hospital experienced PADEs.

## Introduction

The World Health Organization (WHO) announced the third Global Patient Safety Challenge as “medication without harm”^1^. The third Global Patient Safety Challenge seeks the commitment of health-care workers, regulatory agencies, researchers, pharmaceutical corporations, and higher institutions. Its goal will be to “reduce the level of severe, avoidable harm related to medications by 50% over 5 years, globally”^2^.

National coordinating council for medication error reporting and prevention defines a medication error as “any preventable event that may cause or lead to inappropriate medication use or patient harm while the medication is in the control of the health care professional and patient”^3^. Medication errors are a significant health burden causative to over half of all ADEs among hospitalized patients^4^. Globally, the price related to medication errors has been 42 billion $/year, not considering lost wages, productivity, or health care prices^5^.

Potential adverse drug event (PADE) is a medication error with the potential to cause associate degree injury however that does not cause any injury, either due to specific circumstances, chance, or as a result of the error is intercepted and corrected^6^. It has been reported that PADEs constitute over 17 million emergency department visits and 8 million hospital admissions per year in the United States^7^. The incidence of PADEs was estimated to be 13.8 per hundred admissions in Saudi Hospital^8^.

Among the 52 medication errors reported in Morocco, 53.8% result in clinically significant potential harm and 46.2% result in actual patient harm. According to this report, there were 7.7 medication errors for a thousand patient-days. The preventable event occurrence was higher in the ordering (71.1%), followed by the administration (21.2%) and transcribing stage (5.7%)^9^.

The incidence of medication errors per thousand patient-days ranges from 7.7 to 40.9^9,10^. Of all medication errors, the prescribing and monitoring were the most common error stages^10^. Seventy-one percent of the potentially harmful medication error occurrence was found to be at the ordering stage of the medication-use process^11^. Non-psychiatric drugs were three times as likely to cause ADEs compared to psychiatric drugs^10^.

The total prescribing error rate was 40.9% with 1.3% significant errors, in Nigeria. Duration of treatment omission and abbreviations which can lead to serious errors was the most common^12,13^. Medication administration errors in a University Hospital in Egypt, about 5531 errors were observed with 2.67 errors per observation and the overall error rate was 37.68%^14^.

In our country, medication error incidence of 56.4%^15^, 40 per 100 orders^16^ and 52.5%^17^ were reported. Wrong drug combination (28.13^16^, 25.7%^17^), wrong frequency (15.5%^17^), omission errors (42.89%^16^) and wrong dose (8.36^16^, 15.1%^17^) were the common medication ordering errors. The medication administration errors were found to be 51.8%; wrong timing (30.3%) and missed doses (18.3%) were the common administration errors^18^. The errors ranged from 16.8 to 28.6% for non-intravenous medications and from 20.6 to 33.4% for intravenous medications^19^.

Hospitalized patients are more likely exposed to polypharmacy. This, in turn, is a concern for PADEs. Patients who have PADEs are likely to have a longer hospital stay, reduced quality of life, increased overall health care cost, and an increased risk of morbidity and mortality. To our knowledge, in Ethiopia, there is no prospective observational study that followed patients admitted in the ward to identity the incidence, severity, preventability of PADEs. Therefore, this study aimed to determine the incidence, contributing factors, predictors, severity, and preventability of PADEs among hospitalized adult patients at Jimma Medical Center.

## Methods

### Study setting and period

The study was conducted among hospitalized patients at the medical ward of Jimma Medical Center (JMC), the only medical center in the south–west part of the country with 800 active beds^20^.

### Study design & population

A prospective observational study was conducted among adult patients admitted to inpatient medical wards or units.

### Participant’s eligibility and inclusion

Participant's eligibility and inclusion were performed according to previous study report^20^. PADEs during/before admission were not included in the calculated incidence.

### Sample size and sampling technique

The sample size equals 319 was calculated based on the assumption detailed in the previous article^20^. The proportion of PADE occurrence (P) = 0.525 was taken from a study done in JMC^18^.

### Data collection instrument, procedures, and quality assurance

A semi-structured questionnaire was designed by reviewing different literature for important variables^3,21–23^. Patient medical chart review, patient interview, and direct observation was performed to obtain the data^20^. PADEs were identified on the conditions that medication errors that can cause clinically serious harm in advance^3^. Drug-drug interaction was assessed as per Lexicomp drug interaction classification since Lexicomp Interactions scored highest in scope and completeness compared to seven drug information resources^24,25^. Drug-drug interaction with major severity levels; contra-indicated (avoid combination) and consider therapy modification were considered. The severity of PADEs was classified according to the National coordinating council for medication error reporting and prevention (NCCMERP) severity category modified definition^21^ and according to the stage in the medication use stages, they have occurred as prescribing (ordering), dispensing, administering, transcribing and monitoring. The training was given to data collectors on the data collection procedure and research objectives. Before exporting to SPSS, data was checked and cleared in EpiData to exclude ambiguous, incomplete, and erroneous data.

### Study variables

#### Dependent variable

PADE occurrence.

#### Independent variables

Patient-related: Age, sex, educational status, residence, marital status, occupation, cigarette smoking, and alcohol consumption. Disease-related: History of previous ADRs, comorbidity (Charlson’s comorbidity index), admission diagnosis, length of hospital stay, previous hospitalization, and previous medical condition. Medication-related: Drug category, number of drugs, traditional medicine use, and history of medication use.

### Outcome measures and validating methods

In the current study, methods used for detecting PADEs include a chart review, patient interview, and direct observation^20^. The patient’s medical chart and documents such as the progress note, laboratory result, prescriber’s orders, and drug administration chart were assessed^20^.

### Data processing and analysis

Statistical Package for Social Sciences (SPSS) version 24 and Microsoft Excel (2010) were used for analysis. Multivariate logistic regression was performed to identify independent predictors of PADE occurrence. A p-value of < 0.05 was considered to be statistically significant.

The outcome of the study was reported as PADEs incidence per 100 admissions, per 1000 patient-days, and per 100 medication orders; severity of PADEs; the percentage of PADEs in stages of medication use (ordering/prescribing, transcribing, dispensing, administering, or monitoring).

- “PADEs incidence per 100 admissions: The total number of PADEs identified, divided by the total number of admissions; multiplied by 100”
- “PADEs incidence per 1000 patient-days: The total number of PADEs identified, divided by the total number of patient- days multiplied by 1,000”
- “PADEs incidence per 100 medication orders: The total number of PADEs identified, divided by the sum of medications ordered multiplied by 100”

### Ethical approval and consent to participate

Ethical clearance & approval was obtained from the institutional review board (IRB) of Jimma University with the reference number of IHRPGD/550/19. It was based on the 1964 Helsinki declaration and its later amendments or comparable ethical standards. Before the start of the study, written informed consent was requested and received from the patient. Informed consent was obtained from all individual participants included in the study.

### Operational definitions and definition of terms

- Medication errors: “Any preventable event that may cause or lead to inappropriate medication use or patient harm while the medication is in the control of the healthcare professional and patient”^6,26^.
- PADE: “A medication error with the potential to cause an injury but which does not actually cause any injury, either because of specific circumstances, chance, or because the error is intercepted and corrected”^3^.
- Educated: Participants who had primary, secondary, or tertiary education.

## Results

### Socio-demographic characteristics of the study participants

From a total of 319 participants, 158 (49.5%) of them were males. The mean ± SD age of the participants was 43 ± 17.6 years. Most of the participants, 225 (70.5%) were from a rural area. About 27.3% of study participants drunk alcohol and 14 (4.4%) patients had used traditional medicine. The mean ± SD and the total length of hospital stay of the patients were 17.8 ± 14.5 days and 5667 patient-days respectively. Comorbidities were determined by weighted Elixhauser Comorbidity Index (ECI) and the mean ± SD of ECI was 5.7 ± 5.8. The mean ± SD number of medications prescribed for the study participants was 4.4 ± 2 (Table 1).

**Table 1:** Socio-demographic characteristics of study participants.

Variables	Frequency (%) (N = 319)	PADEs (%) (N = 94)
**Sex**		
Male	158 (49.5)	45 (47.9)
**Age(years)**		
Mean ± SD	43 ± 17.6	41.1 ± 16.5
18–35	123 (38.6)	41 (43.6)
36–50	92 (28.8)	28 (29.8)
51–65	67 (21)	17 (18.1)
≥ 66	37 (11.6)	8 (8.5)
**Residence**		
Rural	225 (70.5)	62 (66)
Urban	94 (29.5)	32 (34)
**Educational status**		
Uneducated	218 (68.3)	66 (70.2)
Educated	101 (31.7)	28 (29.8)
Alcohol user	87 (27.3)	21 (22.3)
Cigarette smoker	26 (8.2)	6 (6.4)
Traditional medicine user	14 (4.4)	3 (3.2)
**Number of medications**		
Mean ± SD	4.4 ± 2	5.3 ± 2.2
1–3 drugs	121 (37.9)	22 (23.4)
4–6 drugs	155 (48.6)	47 (50)
≥ 7 drugs	43 (13.5)	25 (26.6)
Had a history of adverse drug reaction(s)	11 (3.4)	4 (4.3)
Had a history of hospitalization in the preceding 3 months	76 (23.8)	30 (31.9)
**Length of hospital stay, days**		
Mean ± SD	17.8 ± 14.5	20.8 ± 16.5
1–7	54 (16.9)	17 (18.1)
8–14	116 (36.4)	28 (29.8)
15–21	67(21)	17 (18.1)
≥ 22	82 (25.7)	32 (34)
**Elixhauser comorbidity index**		
Mean ± SD	5.7 ± 5.8	4.9 ± 4.89
≤ 15	295 (92.5)	91 (96.8)
> 15	24 (7.5)	3 (3.2)

### Diagnosis of study participants

The diagnoses of the patients were categorized according to the international classification of disease (ICD)-10 codes. Most of the patients were diagnosed with diseases of the circulatory system (53%), infectious and parasitic diseases (34.5%), and diseases of the genitourinary system (28.5%). The diagnosis category most commonly associated with PADEs were diseases of the circulatory system (51.1%) and infectious and parasitic diseases (46.8%) (Table 2).

**Table 2:** The diagnosis of study participants.

ICD-10 code	Diagnosis category	Frequency (%) (N = 319)	PADEs (%) (N = 94)
I00-I99	Diseases of the circulatory system	169 (53)	48 (51.1)
A00-B99	Infectious and parasitic diseases	110 (34.5)	44 (46.8)
N00-N99	Diseases of the genitourinary system	91 (28.5)	21 (22.3)
D50-D89	Diseases of the blood and immune mechanism	86 (27)	20 (21.3)
E00-E89	Endocrine, nutritional and metabolic diseases	69 (21.6)	25 (26.6)
G00-G99	Diseases of the nervous system	64 (20.1)	21 (22.3)
K00-K95	Disease of the digestive system	63 (19.7)	23 (24.5)
J00-J99	Diseases of the respiratory system	62 (19.4)	16 (17)
C00-D49	Neoplasms	7 (2.2)	2 (2.1)
L00-L99	Diseases of the skin and subcutaneous tissue	5 (1.6)	0
S00-T88	Injury and other external causes	3 (0.9)	1 (1.1)
F01-F99	Mental and Neurodevelopmental disorders	1 (0.3)	1 (1.1)

Among the patients involved in the study, 171 (53.6%) had a previous medical condition. Diseases of the circulatory system 88 (51.46%), infectious and parasitic diseases 48 (28.07%), and endocrine, nutritional and metabolic diseases 25 (14.62%) were the common previous medical condition of the patients (Table 3).

**Table 3:** Previous medical condition of the study participants.

ICD-10 Code	Diagnosis category	Frequency (%) (N = 171)	PADEs (%) (N = 94)
I00-I99	Diseases of the circulatory system	88 (51.46)	25 (26.6)
A00-B99	Infectious and parasitic diseases	48 (28.07)	19 (20.2)
E00-E89	Endocrine, nutritional and metabolic diseases	25 (14.62)	8 (8.5)
J00-J99	Diseases of the respiratory system	14 (8.18)	2 (2.1)
N00-N99	Diseases of the genitourinary system	12 (7.02)	5 (5.3)
D50-D89	Diseases of the blood and immune mechanism	7 (4.09)	3 (3.2)
K00-K95	Disease of the digestive system	5 (4.63)	0
G00-G99	Diseases of the nervous system	5 (4.63)	2 (2.1)
C00-D49	Neoplasms	2 (1.17)	1 (1.1)

### Admission medication(s)

A total of 1395 medications were prescribed for the study participants. Most of the patients received antibiotics (50.8%), cardiovascular medicines (48.3%), gastrointestinal medicines (35.7%), and analgesics (28.2%). Medication classes most commonly associated with PADEs were antibiotics (55.3%) followed by gastrointestinal medicines (43.6%) and cardiovascular medicines (39.4) (Table 4).

**Table 4:** Types of medication prescribed on admission for study participants.

S.No	Class of medication	Frequency (%) (N = 319)	PADEs, n (%) (N = 94)
1	Antibiotics	162 (50.8)	52 (55.3)
2	Cardiovascular medicines	154 (48.3)	37 (39.4)
3	Gastrointestinal medicines	114 (35.7)	41 (43.6)
4	Analgesics	90 (28.2)	34 (36.2)
5	Vitamins and antianemic agents	78 (24.5)	26 (27.7)
6	Electrolytes	59 (18.5)	13 (13.8)
7	Antiplatelates	54 (16.9)	16 (17)
8	Antidyslipidemic agents	53 (16.6)	18 (19.1)
9	Anticoagulants	52 (16.3)	24 (25.5)
10	Antituberculosis	43 (13.5)	18 (19.1)
11	Steroids	38 (11.9)	13 (13.8)
12	Antidiabetics	27 (8.5)	10 (10.6)
13	Antiseizures	22 (6.9)	18 (19.1)
14	Antivirals	21 (6.6)	11(11.7)
15	Antifungals	12 (3.8)	10 (10.6)
16	Antiasthmatics	11 (3.4)	1(1.1)
17	Anti-thyroid agents	9 (2.8)	4 (4.3)
18	Antipsychotics	9 (2.8)	6 (6.4)
19	Antimalarials	6 (1.9)	2 (2.1)
20	Antihistamines	3 (0.9)	1(1.1)

### Medication history

Based on documented and available data, 166 (52%) patients had a history of medication use in the 3 months before the study period. One hundred eight patients were on medication during admission. Most of the patients were on cardiovascular medicines 79 (73.15%), antibiotics 28 (25.93%), and antiviral agents 28 (25.93%) (Table 5).

**Table 5:** Types of medication history of the study participants.

S.No	Class of medication	Frequency (%) (N = 108)	PADEs, n (%) (N = 94)
1	Cardiovascular medicines	79 (73.15)	24 (25.5)
2	Antibiotics	28 (25.93)	13 (13.8)
3	Antivirals	28 (25.93)	14 (14.9)
4	Antituberculosis	11 (10.19)	1 (1.1)
5	Antiplatelates	11 (10.19)	3 (3.2)
6	Antidyslipidemic agents	10 (9.26)	3 (3.2)
7	Antiasthmatics	10 (9.26)	1 (1.1)
8	GI medicines	9 (8.33)	3 (3.2)
9	Steroids	7 (6.48)	0
10	Antimalarials	6 (5.56)	0
11	Anticoagulants	5 (4.63)	2 (2.1)
12	Antianemic agents	5 (4.63)	2 (2.1)
13	Antiseizures	5 (4.63)	3 (3.2)
14	Antipsychotics	4 (3.70)	3 (3.2)
15	Analgesics	3 (2.78)	0
16	Anti-thyroid agents	2 (1.85)	1 (1.1)

### Incidence of PADEs

**Figure 1:**
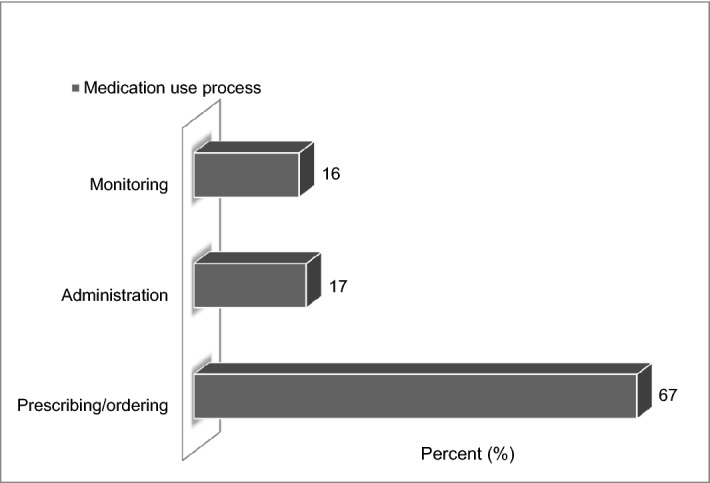
Stages of the medication use process at which PADEs occurred. Microsoft Excel (2010) https://www.microsoft.com/en-us/microsoft-365/previous-versions/microsoft-excel-2010 was used to generate the figure. Key: “(B) An event occurred but the medication did not reach the patient. (C) An event occurred that reached the patient but did not cause harm. (D) An event occurred that reached the patient and required monitoring to confirm that it resulted in no harm to the patient and/or required intervention to preclude harm”.

**Figure 2:**
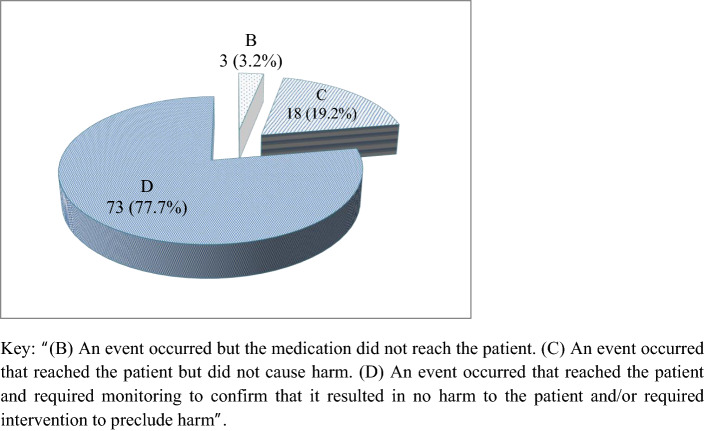
Severity of PADEs. Microsoft Excel (2010) https://www.microsoft.com/en-us/microsoft-365/previous-versions/microsoft-excel-2010 was used to generate the figure.

A total of 94 PADEs were identified during the 3 months of the study period. The incidence of PADEs were 29.47 (95% CI: 23.8–36.06) per 100 admissions, 16.59 (95% CI: 13.55–20.3) per 1000 person-days, and 6.74 (95% CI: 5.45–8.25) per 100 medication orders. PADEs were occurred at prescribing 63 (67%), administration 16 (17%), and monitoring 15 (16%) stages (Fig. 1); and all are preventable by definition. The severity of PADEs was assessed by the NCC MERP severity category. Accordingly, 73 (77.7%) were category D, 18 (19.2%) were category C and 3 (3.2%) were category B (Fig. 2). The clinical pharmacists and clinical pharmacy postgraduate students working in the ward intervened and prevented the PADEs from causing harm.

### Factors associated with the occurrence of PADEs

In univariate analysis, factors associated with PADEs were analgesics, antiviral agents, anticoagulants, anti-seizures, cardiovascular medicines, number of medications, ECI ≤ 15 and previous hospitalization in the past 3 months. The number of medications, ECI, anti-seizures, anti-TB agents, and anticoagulants were independent predictors of PADEs.

Patients who received ≥ 7 medications were 5.1 times more likely to experience PADEs when compared to patients who received ≤ 3 drugs (AOR = 5.12; 95% CI: 2.01–13.05; p = 0.001). Patients with ECI ≤ 15 were 6.2 times more likely to experience PADEs compared to patients with ECI > 15(AOR = 6.24; 95%CI: 1.48–26.25; p = 0.013). Patients who were on anticoagulants were about 2.5 times more likely to develop PADEs than those who were not on anticoagulants (AOR = 2.51; 95%CI: 1.22–5.19; p = 0.013). Patients receiving anti TB were 2.2 times more likely to develop PADEs than who were not on anti TB (AOR = 2.15; 95%CI: 1.002–4.59; p = 0.049). Patients who were on anti-seizure were 22 times more likely to develop PADEs than those who were not on anti-seizures (AOR = 21.96; 95%CI: 6.57–73.39; P < 0.0001) (Table 6).

**Table 6:** Factors associated with PADE occurrence.

Variables	PADEs occurrence		Total n (%)	COR (95% CI)	P-value	AOR (95% CI)	P-value
	No; n (%)	Yes; n (%)					
**Residence**							
Rural	163 (51.1%)	62 (19.4%)	225 (70.5%)	1		1	
Urban	62 (19.4%)	32 (10%)	94 (29.5%)	1.357 (.809–2.276)	0.247	1.141 (0.599–2.174)	0.688
**Previous hospitalization**							
No	179 (56.1%)	64 (20.1%)	243 (76.2%)	1		1	
Yes	46 (14.4%)	30 (9.4%)	76 (23.8%)	1.824 (1.06–3.134)	0.030	1.856 (0.975–3.534)	0.06
**Alcohol consumption**							
No	159 (49.8%)	73 (22.9%)	232 (72.7%)	1		1	
Yes	66 (20.7%)	21 (6.6%)	87 (27.3%)	0.693 (.394–1.218)	0.202	0.500 (0.25–1.00)	0.05
**Number of medications**							
1–3 drugs	99 (31%)	22 (6.9%)	121 (37.9%)	1		1	
4–6 drugs	108 (33.9%)	47 (14.7%)	155 (48.6%)	1.958 (1.102–3.48)	0.022	1.844 (0.978–3.48)	0.059
≥ 7 drugs	18 (5.6%)	25 (7.8%)	43 (13.5%)	6.25 (2.917–13.39)	P < 0.0001	5.119 (2.007–13.053)	0.001
**Elixhauser comorbidity index (ECI)**							
> 15	21 (6.6%)	3 (0.94%)	24 (7.52%)	1		1	
≤ 15	204 (63.95%)	91 (28.53%)	295(92.5%)	3.123 (0.908–10.733)	0.071	6.239 (1.483–26.25)	0.013
**Length of hospital stay**							
1–7 days	37 (11.6%)	17 (5.3%)	54 (16.9%)	1	0.124	1	
8–14 days	88 (27.6%)	28 (8.8%)	116 (36.4%)	0.693 (0.34–1.415)	0.314	0.496 (0.212–1.158)	0.105
15–21 days	50 (15.7%)	17 (5.3%)	67(21.0%)	0.74 (0.334–1.639)	0.458	0.482 (0.19–1.24)	0.131
≥ 22 days	50 (15.7%)	32 (10.0%)	82 (25.7%)	1.393 (0.674–2.88)	0.371	0.682 (.277–1.682)	0.406
**Genitourinary system disease**							
No	155 (48.6%)	73 (22.9%)	228 (71.5%)	1		1	
Yes	70 (21.9%)	21 (6.6%)	91 (28.5%)	0.637 (0.36–1.117)	0.115	1.108 (0.546–2.249)	0.78
**Blood & immune disease**							
No	159 (49.8%)	74 (23.2%)	233 (73%)	1		1	
Yes	66 (20.7%)	20 (6.3%)	86 (27.0%)	0.65 (0.368–1.153)	0.141	0.549 (0.274–1.103)	0.092
**Endocrine & metabolic disease**							
No	181 (56.7%)	69 (21.6%)	250 (78.4%)	1		1	
Yes	44 (13.8%)	25 (7.8%)	69 (21.6%)	1.49 (0.848–2.619)	0.165	1.43 (0.702–2.91)	0.324
**Digestive system disease**							
No	185 (58%)	71 (22.3%)	256 (80.3%)	1		1	
Yes	40 (12.5%)	23 (7.2%)	63 (19.7%)	1.498 (0.838–2.68)	0.173	1.285 (0.62–2.67)	0.503
**Antivirals**							
No	215 (67.4%)	83 (26%)	298 (93.4%)	1		1	
Yes	10 (3.1%)	11 (3.4%)	21 (6.6%)	2.849 (1.167–6.96)	0.022	2.73 (0.96–7.72)	0.059
**Anticoagulants**							
No	197 (61.8%)	70 (21.9%)	267 (83.7%)	1		1	
Yes	28 (8.8%)	24 (7.5%)	52 (16.3%)	2.412 (1.31–4.438)	0.005	2.51 (1.22–5.19)	0.013
**Anti-tuberculosis agents**							
No	200 (62.7%)	76 (23.8%)	276 (86.5%)	1		1	
Yes	25 (7.8%)	18 (5.6%)	43 (13.5%)	1.895 (0.978–3.67)	0.058	2.15 (1.002–4.59)	0.049
**Gastro-intestinal medicines**							
No	152 (47.6%)	53 (16.6%)	205 (64.3%)	1		1	
Yes	73 (22.9%)	41 (12.9%)	114 (35.7%)	1.611 (0.983–2.64)	0.059	1.16 (0.58–2.33)	0.67
**Cardiovascular medicines**							
No	108 (33.9%)	57 (17.9%)	165 (51.7%)	1		1	
Yes	117 (36.7%)	37 (11.6%)	154 (48.3%)	0.599 (0.367–0.98)	0.040	0.86 (0.43–1.72)	0.669
**Anti-seizures**							
No	221 (69.3%)	76 (23.8%)	297 (93.1%)	1		1	
Yes	4 (1.3%)	18 (5.6%)	22 (6.9%)	13.086 (4.29–39.9)	P < 0.0001	21.96 (6.57–73.39)	P < 0.0001
**Analgesics**							
No	169 (53%)	60 (18.8%)	229 (71.8%)	1		1	
Yes	56 (17.6%)	34 (10.7%)	90 (28.2%)	1.71 (1.019–2.871)	0.042	1.37 (0.74–2.56)	0.32
**History of medication use in the preceding 3 months**							
No	114 (35.7%)	39 (12.2%)	153 (48%)	1		1	
Yes	111 (34.8%)	55 (17.2%)	166 (52%)	1.448 (0.89–2.356)	0.136	0.84 (0.41–1.73)	0.63

*AOR* adjusted odds ratio, *COR* crude odds ratio, *CI* confidence interval, *ECI* elixhauser comorbidity index.

**Figure 3:**
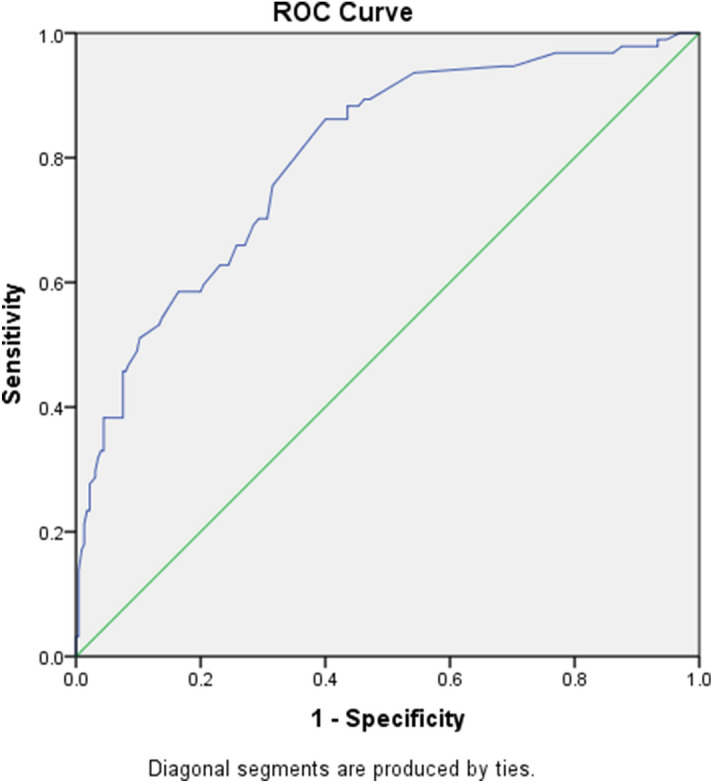
Receiver operating characteristic curve for the PADE occurrence. The area under the curve was 0.802 (95% CI: 0.749–0.854). The straight diagonal line represents reference (no discriminative ability). IBM SPSS Statistics for Windows, Version 24.0. Armonk, https://www.ibm.com/analytics/spss-statistics-software was used to generate the figure.

Area under the receiver operating characteristics (AUROC) = 80.2% (95%CI: 74.9%- 85.4%) (Fig. 3).

## Discussion

Medication errors may occur at any medication use stages (prescribing, dispensing, administration, and monitoring) and can result in severe harm, disability, and even death which are avoidable harm^27^. Health care systems should design specific programs of action for improving patient safety in each of four medication use stages, developing strategies, plans, and tools to ensure that the medication process has the safety of patients, monitoring medication-related harm, and producing a strategy for setting out research priorities^2^.

In present study, the incidence of PADEs were 29.47 (95% CI 23.8–36.06) per 100 admissions (crude rate), 16.59 (95% CI 13.55–20.3) per 1000 person-days and 6.74 (95% CI 5.45–8.25) per 100 medication orders. This is comparable with a study in Saudi Arabia^28^, 16.9 (95% CI 15.7 to 18.3) per 100 admissions, 21.8 (95% CI 20.2 to 23.5) per 1000 person-days. The higher incidence rate was observed in the current study compared to 5.5 PADEs per 100 admissions reported by Bates and colleagues^29^.

Multivariate analysis indicated that the number of medications the patient was receiving, ECI, anti-seizures, anti-TB agents, and anticoagulants were independent predictors of PADE occurrence. The ability of these variables to predict PADE occurrence was assessed using AUROC, which is 80.2% (95% CI: 74.9–85.4%); thus the model demonstrated excellent performance.

Patients who received greater than or equal to 7 medications had higher odds of experiencing PADEs among the study participants. In line with this, Diaz and colleagues^30^ reported an increased number of prescribed medications were significantly associated with all adverse events. Using multiple drugs concurrently, ADEs result from alterations of the pharmacokinetics parameters^31^.

Anticoagulants were independently associated with the occurrence of PADEs. In anticoagulant therapy, the thrombotic and hemorrhagic risk is easily affected by factors such as age, co-morbidities, and concomitant medications. PADEs of anticoagulants are influenced by the types of anticoagulant agents, therapeutic versus prophylactic therapy, and duration of treatment^32^. Besides, anticoagulants have a narrow therapeutic index, and pharmacokinetics or pharmacodynamics interactions with other drugs may result in PADEs^33^.

Anti-seizures were significantly associated with the occurrence of PADEs. When other drugs combined with anti-seizures to treat intercurrent illness, there is a probability of PADEs, because anti-seizures are commonly given for prolonged time, have a narrow therapeutic window, and little alterations in their pharmacokinetics can result in toxic effects. Carbamazepine, phenytoin, valproic acid, and phenobarbital greatly alter liver enzymes and can affect the metabolism of other combined medications^34^.

Anti-TB was also found to have a significant association with the occurrence of PADEs. Rifampin, isoniazid, and pyrazinamide are hepatotoxic and their interaction with other drugs will increase the risk of PADEs. Genetic causes, advanced age, malnutrition, high dosage, and multiple comorbidities are predisposing factors for PADEs of anti-TB agents^35^.

Patients who were receiving antiviral agents were more likely to experience PADEs than patients who were not receiving these agents. Mok and colleagues^36^ noted a significant number of PADEs of antiviral agents, leading to severe PADEs. Anwikar and colleagues^37^ observed a highly significant association between the use of zidovudine and anemia.

## Conclusion

The incidence of PADEs was 29.47 per 100 admissions, 16.59 per 1000 person-days, and 6.74 per 100 medication orders. The most common stage of the medication use process at which PADEs occurred was at the prescribing stage. The number of medications, ECI, anti-seizures, anti-TB agents, and anticoagulants were independent predictors of the occurrence of PADEs.

## Data Availability

The data sets generated during and/or analyzed during the current study are available from the corresponding authors on reasonable request.

## Abbreviations

ADE: Adverse drug event
ADR: Adverse drug reaction
AOR: Adjusted odds ratio
AUROC: Area under the receiver operating characteristic
ECI: Elixhauser comorbidity index
ICU: Intensive care unit
JMC: Jimma Medical Center
LOS: Length of stay
NCCMERP: National Coordinating Council for Medication Error Reporting and Prevention
WHO: World Health Organization
